# Inflammatory Choroidal Neovascularization

**DOI:** 10.4103/0974-9233.58422

**Published:** 2009

**Authors:** Piergiorgi Neri, Marta Lettieri, Cinzia Fortuna, Mara Manoni, Alfonso Giovannini

**Affiliations:** 1 The Eye Clinic-Ospedali Riuniti Umberto I-G.M. Lancisi-G. Salesi-Ancona,; 2 The Eye Clinic-Ospedali Riuniti Umberto I-G.M. Lancisi-G. Salesi-Ancona; Polytechnic University of Marche, Ancona, Italy

**Keywords:** Choroidal Neovascularization, Choroiditis, Immunosuppression, Steroids, Uveitis, Vascular Endothelial Growth Factor

## Abstract

**Purpose and Methods:**

Choroidal neovascularization (CNV) can be a severe sight-threatening sequela, which can be secondary to both infectious and noninfectious uveitis. This review summarizes the different diseases associated with CNV, highlighting new treatment modalities and the possible strategies, which could be applied for the therapy of this occurrence.

**Results:**

Since CNV can often originate from posterior pole lesions and can be hard to identify, an accurate examination is mandatory in order to identify the correct diagnosis. In the majority of cases, fluorescein angiography (FA), indocyanine green angiography (ICGA) and optical coherence tomography (OCT) enable the determination of the clinical characteristics of the CNV. An infectious disease should be looked for to include a suitable therapy when available. The treatment strategy for CNV secondary to noninfectious uveal inflammations should be directed at controlling the inflammatory process. Systemic corticosteroids with or without immunosuppressive agents are indicated even when the CNV occurs with apparently inactive uveitis: Chronic subclinical inflammation can be the basis for the pathogenesis of CNV. Additional therapies aimed directly at the neovascular process, such as the intravitreal anti-Vascular Endothelial Growth Factor (VEGF) agents, are recommended particularly when the therapy shows an insufficient response.

**Conclusion:**

CNV secondary to uveitis is a severe sequela leading to significant visual impairment. ICGA is mandatory in order to obtain relevant information about the choroidal status. Several therapeutic options have been considered, but no guidelines are provided at the moment. Moreover, the current data are still only based on case reports or small series. For such reasons, further trials are mandatory to validate the preliminary available results.

## INTRODUCTION

Choroidal neovascularization (CNV) is one of the most severe causes of visual impairment in patients with uveitis. When the disruption of the homeostasis between the retinal pigment epithelium (RPE) and Bruch's membrane occurs, a vicious circle leads to the choroidal neo-angiogenesis. Both noninfectious and infectious uveitis can have CNV as a possible sequela: Infectious uveitis is due to infective agents that directly affect the retino-choroidal space whereas noninfectious uveitis is likely to be an autoinflammatory disease.

The imbalance between inhibitory^1^ and stimulatory^2^ action of the soluble mediators produced by the RPE^3^  is supposed to be the trigger of the neoangiogenesis.

The animal models^4^  proved that neovessels occur, even if inflammation is not evident.

Often, biomicroscopy and fluorescein angiography (FA) may not be sufficient to explore the choroidal status: Only choroidal foci of significant size causing alterations of the retina red reflex can be detected through the screen of the RPE by funduscopy and/or FA.

For these reasons, indocyanine green angiography (ICGA) is mandatory to show choroidal abnormalities. Choroidal inflammation was reclassified according to the structures that are predominantly involved in the inflammatory process due to ICGA. Since choriocapillaris non-perfusion involve the chorioretinal interface, the RPE and outer retina, the production of inflammatory mediators induces the VEGF release and, consequently, the CNV pathogenesis.

Choroidal neovessels grow into the sub-RPE space or into the subretinal space.^5^  The location, the growth pattern and the type of CNV depend on the patient's age and the underlying disease, albeit uveitic CNV is typically the type 2. Bleeding and exudation can occur with further growth, accounting for the visual symptoms.

As previously stated, CNV can be the sequela of both infectious and noninfectious uveitis; in the infectious diseases, Toxoplasmosis,^6^  Toxocara canis,^7^  Tuberculosis,^8^  viral retinopathies,^9^  can have CNV as synchronous or asynchronous sequela.

As well as infectious uveitis, noninfectious uveitis has been associated with CNV also; punctate inner choroidopathy (PIC), multifocal choroiditis (MFC), acute posterior multifocal placoid pigment epitheliopathy (APMPPE) and Vogt-Koyanagi-Harada (VKH) disease are some of the disorders that are associated with CNV occurrence.

In the following sections the major uveitis issues will be reviewed; posterior infectious and noninfectious uveitis entities most often associated with CNV will be reported.

## CHOROIDAL NEOVASCULARIZATION SECONDARY TO INFECTIOUS UVEITIS

Many protozoa can infect the eye: Trypanosome,^10^  leishmania^11^  and pneumocystis carinii^12^  have been described as possible parasites affecting the retina. Toxoplasma gondii 6  is the protozoan that most commonly affects the eye and the only one that has been associated with CNV occurrence. Toxoplasma gondii belongs to the genus Toxoplasma and is a ubiquitous, endocellular parasite in humans.^13^–^15^

Until recently congenitally acquired toxoplasmosis was thought to make up for the majority of toxoplasmic retinochoroiditis, at this time, the acquired disease seems to be the most common type of ocular toxoplasmosis.^16^,^17^

**Figure 1:**
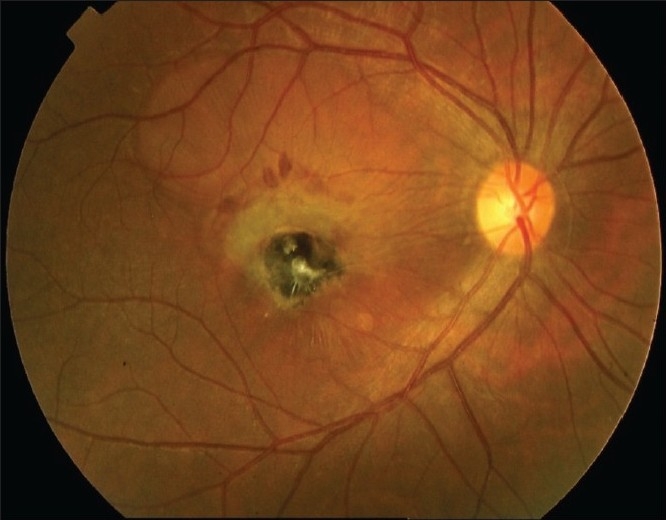
Choroidal neovascularization secondary to inactive toxoplasmosis. Note the fresh blood surrounding the edge of the neovascular membrane

Association between toxoplasmic retinochoroiditis and CNV is relatively frequent.^6^  CNV typically grows close to the edge of an atrophic chorioretinal scar, although CNV can rarely be synchronous with an active disease. In such cases, FA is always useful to prove the CNV, in order to distinguish the neovascular lesion from the reactivation of retinochoroiditis. The peculiar angiographic findings of the choroiditis are early hypofluorescence and late staining for the atrophic areas and late hypofluorescence when pigment clumps are present. The adjacent CNV has the typical dirty gray appearance on biomicroscopy [Figure 1 ], presenting an early hyperfluorescence and late leakage on FA; in very rare cases, CNV can occur concomitantly with a reactivation of retinochoroiditis.

Optical coherence tomography (OCT) can play a role in order to appreciate the CNV^18^  and can show fluid within the adjacent or overlying retina.

As far as treatment is concerned, in the rare cases where CNV is associated with recurrent retinochoroiditis, the classical association of anti-toxoplasmic antibiotics with corticosteroids should be given.^19^

**Figure 2:**
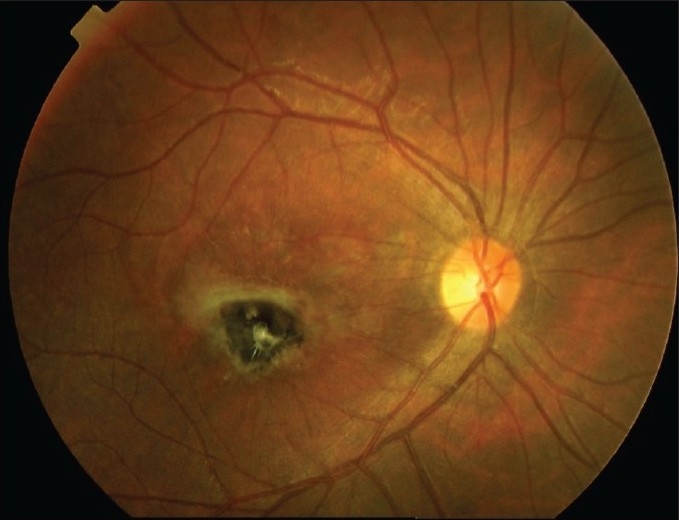
After one month, the membrane showed an evident scarring subsequent to one intravitreal anti-vascular endothelial growth Factor injection

The available options for the treatment of CNV associated with toxoplasmic retinochoroiditis were limited to laser therapy 20  and photodynamic therapy (PDT)^21^  in the recent past. Actually, anti-vascular endothelial growth factor (VEGF)^22^  therapy seems to offer the better outcome and is considered the most reasonable option for such disease [Figure 2 ].

The bacteria can also affect the eye, and CNV can rarely be one of the most severe sequela.^23^–^32^  The retinal colonization can occur by metastasizing the choroid during endocarditis, aortic valve infection, renal and bone abscess and intravenous drug abuse.

Classic CNV is generally the typical occurrence in bacterial endocarditis, which grows close to the primary chorioretinal lesion, or in neighboring area of an old atrophic scar.

Recently, viruses have been associated with CNV, predominantly as a late complication.^33^–^36^

The most recent virus associated with CNV is the West Nile virus: In 2006, Khairallah *etal* .^37^  described a case with an extensive ischemic capillaropathy in the macula which, a few months later, developed a choroidal neovascularization near a chorioretinal scar.

Although most types of fungi, such as Candida albicans, Cryptococcus neoformans and Aspergillus fumigatus, have been described as potential pathogen for the eye; rarely CNV can be a late sequela and no treatment strategies are available at the moment.

Many parasitic worms, called helminthes, can potentially affect the eyes, although Toxocara canis only may rarely provoke choroidal lesions leading to CNV. The choroidal neovascular membrane typically grows near an active or quiescent choroidal granuloma. FA typically shows hyperfluorescence and late leakage of the dye, while the ICGA can demonstrate an occult CNV underlying the area close to the granuloma.^38^

The majority of infectious uveitis associated with CNV, except ocular toxoplasmosis, are generally reports or small case series and no guidelines are provided on a possible therapeutic strategy.

The therapy is based on the association of systemic drugs and other techniques, such as argon laser photocoagulation, PDT, surgical removal and intravitreal anti-VEGF drugs. On the basis of the data emerging from the anecdotal cases recently described, intravitreal anti-VEGF drugs seem to be the best option with or without other systemic medications; they may offer a less aggressive control of the CNV, reducing the risk of exuberant scarring and optimizing the outcome of the disease.

## NONINFECTIOUS UVEITIS AND CHOROIDAL NEOVASCULAR MEMBRANE

Noninfectious uveitis can affect the posterior pole leading to severe sequela and threatening the visual function.

CNV is commonly associated with MFC, which is found in 32 to 46% of patients.^39^–^41^  In 1973, Nozik and Dorsch reported^42^  two cases resembling POHS, which were characterized by multifocal choroidal spots and panuveitis. More than 10 years later, Dreyer and Gass described 28 additional cases w	ith anterior uveitis, vitritis and multiple lesions in the posterior pole, which they called ‘multifocal choroiditis and panuveitis’. At this time, this disease is commonly called multifocal choroiditis, since the panuveitis is not usually present. CNV secondary to MFC is generally located anterior to the RPE, in the macular region or in the peripapillary area. It has been postulated that CNV is very frequent in MFC because of the widespread involvement of the choriocapillaris.

CNV secondary to MFC is more frequent in inflamed areas, albeit this may originate from an old chorioretinal scar also. Low-grade chronic inflammation can be the core of this process and ICGA frequently shows large areas of non-perfusion indicating ischemia, which may be the trigger of angiogenesis. In such cases, ICGA is essential in the evaluation of the choroidal status.^43^

Although few studies have been published, OCT can play a role; this technique may study the position of CNV with reference to the RPE and can detect fluid in the overlying or adjacent retina.

PIC is another subtype of multifocal choroiditis, which presents multiple choroidal spots and, often, CNV.

Active, yellowish spots can be characterized by an overlying serous detachment of the neurosensory retina,^44^  corresponding to those areas where the patient complains of scotomas and/or metamorphopsia. Choroidal spots can change into faded chorioretinal lesions or atrophic chorioretinal scars. Yellowish lesions tend to depigment and to become yellow-whitish, surrounded by a hyperpigmented edge. CNV can originate near the border of focal chorioretinal scars.

CNV is a common sequela of PIC; the occurrence is estimated at 17 to 40% of eyes, leading to severe and permanent visual impairment.^44^,^45^  As in MFC, ICGA is mandatory to prove choroidal involvement.^46^

In 1932, Junius^47^  introduced the term ‘peripapillary retinochoroiditis’ to describe a posterior pole intraocular inflammation, presenting a serpiginous pattern. Several reports added more cases in the following years,^48^,^49^  and serpiginous choroiditis (SC) became a distinguished entity.

Serpiginous choroiditis is a rare, severe, recurrent and, usually, bilateral disease, which is thought to primarily involve the choriocapillaris, and secondarily the RPE and the rest of the choroid.^47^  The disease typically starts from the peripapillary area and progresses centrifugally to the macula, assuming the typical serpiginous pattern. CNV is a well-known complication of SC in 10 – 25% of affected patients,^50^–^53^  generally occurring close to the edge of chorioretinal lesions.

FA is mandatory to detect the CNV, since this cannot be easily recognized during the active stage of the disease.

Since FA usually underestimates the choroidal involvement, ICGA is needed in order to evaluate choroidal status and for the longitudinal management of SC. Indocyanine green angiography allows far better delineation of subretinal lesions than corresponding fluorescein frames, showing subclinical abnormalities of the choroid appearing as hyperfluorescent areas in the late phases of ICGA with a weak leakage.^54^

One of the most peculiar types of multifocal choroiditis is the presumed ocular histoplasmosis syndrome (POHS). Although the trigger of choroiditis is an infectious disease due to the Histoplasma Capsulatum, POHS seems to be an immune reaction to the pathogen affecting the retina. POHS is characterized by disciform macular detachment associated with peripheral chorioretinal scars and peripapillary atrophy, which are the classical triad of this disease.^55^  The primary cause of visual impairment in POHS is the occurrence of CNV in the macula, which results in exudation and subsequent scarring. Since the disease can be identified as an autoinflammatory disorder, Dees *etal* .^55^  reported the use of immunosuppressive agents for the control of CNV secondary to POHS; patients were treated with the association of steroids, cyclosporine A and, in some cases, azathioprine, achieving a good control in all cases considered.

Even though the diseases mentioned above are more frequently associated with CNV, theoretically, any posterior uveitis can be complicated by choroidal neoangiogenesis. Subretinal fibrosis and uveitis syndrome^56^  (SFU), APMPPE,^57^  birdshot retinochoroidopathy,^58^  multiple evanescent white dots syndrome (MEWDS),^59^,^60^  VKH,^61^  sympathetic ophthalmia (SO)^62^  and Sarcoidisis^63^  are some of the noninfectious uveitis that can present CNV as a possible sequela. Similarly to infectious uveitis, Type 2 membranes are the most common neovascular subset occurring in noninfectious uveitis.

The subtle choroidal inflammation can be the promoter of the neoangiogenic process, even for those CNVs, which apparently have not an evident choroidal inflammation. This is the case of the so-called idiopathic choroidal neovascularization (ICNV), where the CNV is the only reliable finding in the retina and no other abnormalities are detectable. FA is not contributory in understanding a clinical pattern, which can be evocative of posterior pole ocular inflammation. The role of inflammation in the pathophysiology of ICNV has been hypothesized on the basis of ICGA,^64^  further supported by the preceding publications, which showed CNV as a possible sequela.^4^  The supposed role of inflammation in ICNV is important for the treatment strategy, more than in other CNVs, ICNV should be treated as an inflammatory neovascularization.^65^

The strategy for the control of noninfectious uveitic CNV is evolving, although the core of the therapy is still based on the suppression of the triggering inflammation. The histopathological features, the pathophysiology and the preliminary results of some studies suggest a relatively unique method for the management of all the subtypes of CNV secondary to noninfectious uveitis.^66^

Since no guideline for the management of inflammatory CNV is provided, there is not a generally agreed method to treat uveitic CNV. All the proposed techniques present the results as safe and effective, but it is not clear which one could offer more advantages; no randomized control trials are available on the direct comparison between different therapies, and no trials on CNVs secondary to a specific uveitis entities are available.

Several techniques have been proposed for the management of CNVs, such as laser photocoagulation,^67^  periocular and systemic steroids,^68^  PDT, 69,70  immunosuppression^55^  and surgical removal.^71^

Since the inflammatory process is not only loco-regional and the whole immune system appears to be involved,^72^  the use of systemic steroids should be always considered.

**Figure 3:**
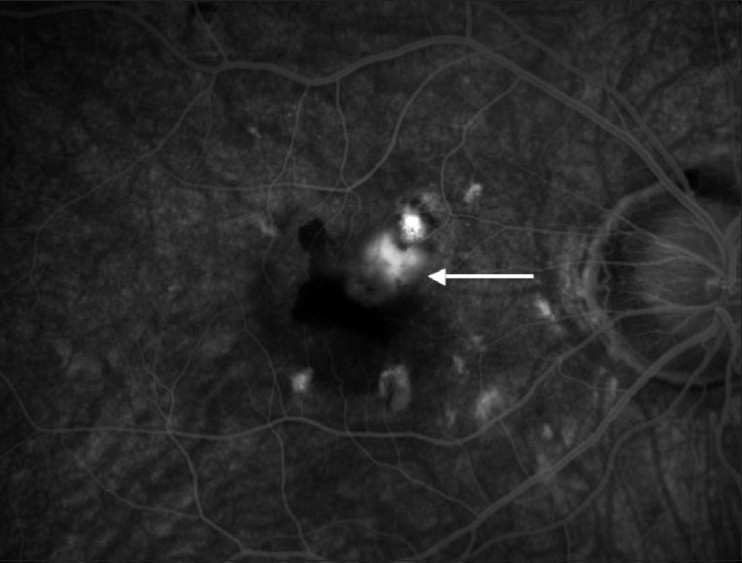
Active juxta/sub-foveal choroidal neovascular membrane, showing leakage at the late phase of the fluorescein angiography (white arrow)

**Figure 4:**
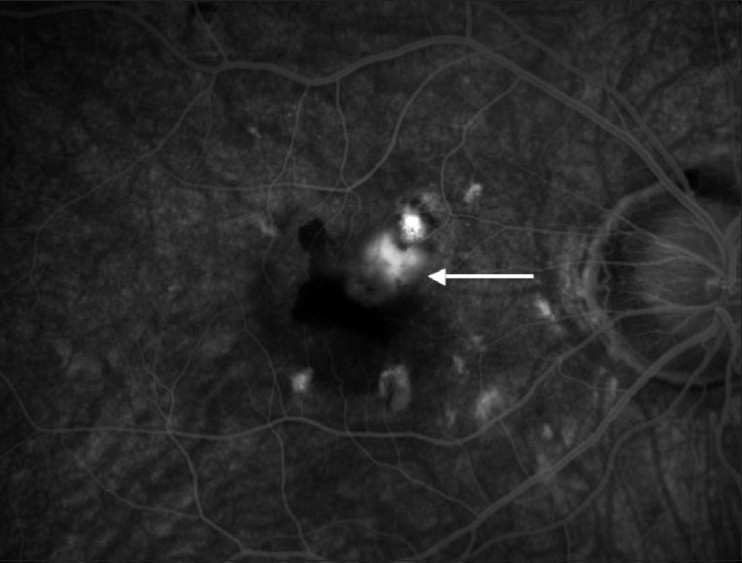
After oral steroids (1 mg/kg) gradually tapered, associated with immunosuppression, the neovascular membrane showed an evident staining in the late phase of the angiogram as well as an evident reduction of its size (white arrowhead)

The safety and efficacy of immunosuppression 55  for the control of choroidal new vessels in uveitis have been described [Figure 3 and 4] . The choice of the immunosuppressant should be established on the basis of the characteristics of the drug itself; some immunosuppressive drugs, such as cyclosporine A,^73^  FK506 and Sirolimus^74^  are known to produce nephrotoxicity, inducing the overexpression of soluble mediators that have an important role in CNV pathogenesis.^75^  Recently, mycophenolate mofetil (MMF) has proven to be effective in reducing such bio-mechanisms,^76^  improving arteriolopathy and decreasing the amount of soluble mediators involved in CNV pathophysiology. For such reasons, MMF can be a promising drug for the long-term control of inflammatory CNV.^77^

When PDT was introduced, the laser treatment deeply changed, limiting argon laser photocoagulation to extrafoveal neovascular membranes to reduce the risk of iatrogenic damage. PDT has been used following different strategies; some patients have been treated electively with medical therapy and PDT,^69^  while others received the PDT when they did not achieve the control of CNV with other strategies.^70^  At this time, PDT has only a marginal role and the new anti-VEGF drugs have a prominent position in the management of inflammatory CNV.^78^

The same is true for surgical removal of CNV; after the introduction of intravitreal anti-VEGF drugs,^78^  surgery is indicated only for extensive peripapillary membranes,^79^  albeit such technique was reported previously as safe and effective.^71^

Although there are no direct comparisons between different treatments, the rationale may suggest medical treatment as first choice for juxta/subfoveal CNV and, when this fails in controlling the CNV activity, anti-VEGF intravitreal drugs should be considered.

## CONCLUSION

Uveitic CNV is a rare but severe complication of uveal inflammation. In most cases the clinical findings obtained by FA, ICGA and OCT allow to establish accurately the characteristics of the CNV. In case of active inflammation, an infectious disease should be looked for to include a suitable therapy when available. Regarding noninfectious uveitis, the treatment strategy should be aimed at controlling inflammation with the support of steroids, usually given systemically but sometimes also locally in unilateral disease, and/or immunosuppressants. Corticosteroids with or without immunosuppressants are suggested also for those CNVs associated with apparent inactive inflammatory disease; subclinical inflammation can be a subtle trigger and can generate the conditions for the occurrence of CNV.^78^,^79^  However, additional therapies aimed directly at the neovascular process are recommended, particularly in severe cases; if steroids with or without immunosuppressive therapy show an insufficient response, anti-VEGF therapy should be rapidly introduced. Argon laser photocoagulation should probably be avoided even in CNV outside the fovea, because intravitreal administration of anti-VEGF therapy is more effective and offers a better long-lasting action. Similarly, PDT seems to be no match to anti-VEGF drugs and recent evidences direct the clinician to use these new types of treatments. However, as for all other methods evaluated, these results are still only based on case reports or small series. For the reasons above, further trials possibly multicentric, randomized and controlled are mandatory to validate these preliminary results.
